# Contrasting perspectives on the risks of intensive livestock farming in The Netherlands: a survey study

**DOI:** 10.1080/13669877.2023.2231003

**Published:** 2023-07-13

**Authors:** V. Eijrond, L. Claassen, D. Timmermans

**Affiliations:** a Department of Public and Occupational Health, Amsterdam UMC, Vrije Universiteit Amsterdam, Amsterdam Public Health Research Institute, Amsterdam, The Netherlands; b Centre for Environmental Security and Safety, National Institute for Public Health and The Environment, Bilthoven, The Netherlands

**Keywords:** Risk perception, mental models, intensive livestock farming, risk communication

## Abstract

In the Netherlands, intensive livestock farming is a recurrent topic of societal debate with stakeholders having quite different perspectives on the benefits and harms. In particular, stakeholders appear to have different perceptions on the risks to human and animal health. This paper reports a quantitative analysis of a survey on the perceptions of risks and benefits of intensive livestock farming conducted among the general public, including people living in livestock dense municipalities (*n* = 808), farmers (*n* = 237) and other stakeholders (*n* = 367). Results show that farmers and citizens have contrasting views about the benefits and concerns and in particular about the risks of intensive livestock farming for human health as well as animal well-being. People living in livestock dense communities held a somewhat more positive view than the general public, yet odour hinder and air quality was perceived as a serious health problem, but not by farmers. These differences in risk perceptions may well be explained from differences in interest, experience and options for control of potential hazards. Our study reflects more than just the perceived risks related to intensive livestock farming, but also reveal the global and multidimensional legitimate concerns and views on what matter to different groups of people. We argue that these differences in risk perspectives should be taken into account when communicating about human health risks, and should also be more explicitly addressed in discussions about the risks of intensive livestock farming in order to develop more inclusive policies that are supported by stakeholders.

## Introduction

In the Netherlands, intensive livestock farming (ILF) is a recurrent topic of societal debate. It can be described as a systemic risk: risk to human health is embedded in a wide-array of concerns in the animal, environmental and economic domains (Kraaij-Dirkzwager, Van der Ree, and Lebret 2017; Renn, Klinke, and Van Asselt 2011). The rapid expansion of large-scale livestock farms in the Netherlands after the Second World War has provided economic benefits, but also led to growing concerns. Especially from the 1990s onwards, the public debate around livestock farming has dealt with issues such as environmental pollution, landscape impairment, concerns around animal welfare and animal diseases, and human health risks (Breeman, Termeer, and van Lieshout 2013; Van de Kerkhof et al. 2010). The large Q fever outbreak (2007–2011) fueled public discussions about the human health risks even more and the potential health risks of living in the vicinity of livestock farms has received more scientific attention. Large-scale epidemiological studies conducted in the Netherlands have found there is a higher risk of pneumonia for residents living in close proximity to goat farms, yet there remain uncertainties about causal relationships (Hagenaars et al. 2017; Heederik et al. 2011; Ijzermans et al. 2018; Ijzermans et al. 2021; Maassen et al. 2016).

The growing societal attention and concerns, scientific knowledge yet at the same time scientific uncertainty regarding the relationship between human health and intensive livestock farming has led to the establishment of the Dutch consortium Knowledge Platform Livestock and Human Health, with the task to make scientific information about the effects of ILF on human health available to professionals and policy makers. Dissatisfaction with current policies have instigated protests against the expansion of livestock farms in local communities and the establishment of local citizen initiatives (Bokma-Bakker et al. 2011; M. Ruiter 2017). At a national level, environmental and animal welfare organisations take a stance against intensive livestock farming, while farmers launched a series of protests triggered by restricting political proposals.

Previous studies have shown disparities between citizens’ and farmers’ perceptions of animal welfare (Te Velde, Aarts, and Van Woerkum 2002; Vanhonacker et al. 2008) as well as human health risks (Van Asselt et al. 2018). Such disagreements may arise because of differences in knowledge, experiences, interests and values (Boogaard et al. 2011; Te Velde, Aarts, and Van Woerkum 2002; Van Asselt et al. 2018). Researchers as well as the Health Council of the Netherlands (2012) call for early and meaningful involvement of stakeholders in designing and implementing risk mitigation measures (Breeman, Termeer, and van Lieshout 2013; Health Council of the Netherlands 2012, 2018b; Post 2021; Termeer 2018; Van Asselt et al. 2018). Involving stakeholders in decision making can help build trust and create support for policies and measures (Siegrist 2021). In order to design risk communication and policies for risk mitigation that speak to stakeholders’ perspectives of risk and benefits and supports their decision making, it is crucial to have a comprehensive understanding of the perspectives of the risks and benefits of the different stakeholders (Health Council of the Netherlands 2012; Kraaij-Dirkzwager, Van der Ree, and Lebret 2017).

Since intensive livestock farming is a multi-faceted problem affecting many parties, a study comparing multi-stakeholder perspectives and exploring several issues simultaneously is necessary. Knowledge around residents’ and farmers’ perceptions towards the multifaceted risks of ILF is currently lacking. Most studies about perspectives on (intensive) livestock farming in the Netherlands focused on a limited group of people, such as, citizens or farmers; on a single issue, such as, animal welfare (Te Velde, Aarts, and Van Woerkum 2002) or public health risks (van Asselt et al. 2018), and focused on a specific type of livestock such as the sow (Bergstra et al. 2015), poultry (Van Asselt et al. 2018) or dairy husbandry (Boogaard 2009).

There is a need to systematically identify the differences in risk perceptions. A useful tool for analysing and understanding the different perspectives towards risks of hazards of different stakeholders is the mental models approach. Mental model elicitation helps to recognize the knowledge and beliefs (i.e. thoughts and ideas), opinions (i.e. explicitly expressed views), concerns and attitudes in regard to a potential threat (Morgan et al. 2002; Slovic 2016). It involves exploring and identifying relevant differences in scientific experts’ and non-scientific experts’ mental models about the health risks of ILF through interviews and a survey (Morgan et al. 2002). The present study is a follow-up study of qualitative research on the mental models of scientific experts, livestock farmers, neighbouring residents and other stakeholders towards the health risks of ILF (Eijrond et al. 2019; Eijrond, Claassen, and Timmermanset al. 2022). Interviews with residents and farmers revealed a wide array of topics they considered important to discuss in relation to the risks and benefits of ILF. In the present survey study, we investigated the perspectives on the risks and benefits of ILF of a larger representative sample of the general public, farmers and other stakeholders. As nearby residents may have a higher health risk than people not living nearby, they may experience ILF differently from the general public (Hagenaars et al. 2017; Heederik et al. 2011; Ijzermans et al. 2018; Ijzermans et al. 2021; Maassen et al. 2016; Simões et al. 2022). Therefore, we also investigated whether people living in livestock dense municipalities hold different perspectives compared to people who do not live in livestock dense communities.

Our research was guided by two main research questions:

1. How does the general public, farmers and other interested stakeholders differ in their perspectives towards the risks and benefits of ILF for society, human health and animal welfare, risk mitigation and risk regulation?
2. Do people living in livestock dense municipalities have different perspectives on the risks and benefits of ILF compared to people living in other municipalities?

## Materials and methods

### Study samples and procedure

Between October 22nd and November 6th 2020, participants were invited to participate in a survey about their attitudes, beliefs, concerns and opinions regarding intensive livestock farming. The survey was conducted in two distinct samples.

#### Research panel sample

Dutch citizens were recruited through an online research panel (Flycatcher Internet Research, www.flycatcher.eu, ISO 20252 certified). The panel had, at that time of study, an active panel population of approximately 10,000 panel members. Panel members provided active consent for participating in surveys and for the gathering and sharing of their demographic information when they joined the panel. They received credits for partaking in the study, which could be exchanged for gifts. A total of 1427 panel members were invited vie email, of which 808 panel members participated in the ‘panel survey’ (response rate 57%). 274 were participants living in pre-selected livestock-dense municipalities in the South of the Netherlands. More specifically, these included people aged 18 and older living in 24 livestock-dense municipalities in Limburg and Noord-Brabant.^1^ The remaining 534 respondents were sampled from other municipalities in the Netherlands and were stratified by gender, age, education, provincial residence andpost code, to represent a fair reflection of Dutch people aged 18 and older.

#### Stakeholder sample

959 opt-in emails with a survey link were sent to poultry, goat and pig farmers in the Southern part of the Netherlands recruited using a Dutch specialist in agrimarketing and market research. We also invited newsletter members (e.g. researchers, farmers, health and environmental professionals, municipal officers, etc.) of the consortium: Knowledge Platform Livestock and Human Health using a digital newsletter. This letter containing a survey link was sent to 700 newsletter members. The survey link therefore reached 1,659 people of which 604 filled in the survey (response rate 36%).

### Survey design

The survey items were based on five topics (general views towards intensive livestock farming, risks for human health and well-being, risks for animals and nature, risk mitigation and risk regulation) and themes that emerged from residents’ and farmers’ appraisals in a previous qualitative study (Eijrond, Claassen, and Timmermans 2022). The survey consisted of statements assessing the beliefs, opinions and concerns about ILF: (1) general attitudes and concerns towards ILF; (2) benefits and risks of ILF for society; (3) risks of ILF for human health and wellbeing; (4) risks of ILF for animal welfare and nature; and (5) risk mitigation and risk regulation. First, respondents were asked which aspects they associated with the term intensive livestock farming, their general attitudes and beliefs and, whether they were concerned about intensive livestock farming and if so, to specify this in an open question. We supplemented the survey with questions according to recent societal concerns regarding the COVID-19 outbreak and the potential association with intensive livestock farming. Next we assessed the beliefs, opinions and concerns. Beliefs were defined as thoughts and ideas towards ILF. Respondents were asked to what extent the statements fit their beliefs on a 5-point Likert scale ranging from 1 *(certainly not)* to 5 *(certainly).* Opinions were operationalized as explicitly expressed subjectively held beliefs which need not be shared by others (Eagly and Chaiken 1993). We asked respondents to what extent they *agreed* or *disagreed* with statements on a 5-point Likert scale. Concerns were measured by asking to what extent they were concerned with a list of aspects on 5-point Likert scale 1 *(not concerned)* to 5 *(very concerned).* Lastly, a range of socio-demographic information was collected of the participants, which were selected according to the literature on perceptions of livestock farming (Boogaard 2009; van Asselt 2019), such as age, gender, educational level, frequency of meat consumption, donator/member to a nature or animal welfare organisation. Table 1 provides an overview of the topics, constructs, items and answer categories.

**Table 1.** Topics and operationalisation of attitudes, beliefs, opinions and concerns towards ILF.

Topics	Attitudes (A), Beliefs (B), & Concerns (C) Opinions (O)	Survey items (number and example)	Answer categories	Cronbach’s *α*
General attitudes and concerns towards ILF	General attitude towards ILF *(A)*	5 items I think ILF is: Positive – negative Unnecessary – necessary (reverse coding) Acceptable – unacceptable Safe – harmful Bad – good (reverse coding)	Five-point semantic scale: 1. Positive …. 5. Negative	0.969
	Concerns about ILF *(C)*	1 item To what extent are you concerned about ILF?	Five-point likert scale: 1. Not concerned … 5. Very concerned	n.a.
	Positive opinion of farmers *(O)*	5 items Farmers with ILF take sufficient account of their living environment. Farmers with ILF are honest in their manure accounting Farmers with ILF use air scrubbers correctly The recent farmers’ protests are justified The media creates a negative image of farmers with ILF	Five-point Likert scale: 1. Disagree …. 5. Agree	0.913
Benefits and risks of ILF to society	Belief about production for export *(B)*	1 item When I think of intensive livestock farming, I think of: Production for export	Five-point Likert scale: 1. Certainly not …. 5. Certainly	n.a.
	Beneficial for the Dutch economy (*O)*	3 items ILF is important for the Dutch economy ILF is necessary for efficient meat production ILF is necessary for the food supply.	Five-point Likert scale: 1. Disagree …. 5. Agree	0.899
	Harmful for the living environment *(O)*	1 item Intensive livestock farming is at the expense of the living environment	Five-point Likert scale: 1. Disagree …. 5. Agree	n.a.
Risks of ILF for human health & well-being	Concerns about Human Health & Wellbeing *(C)*	3 items To what extent are you concerned about: Human welfare Human health Infectious disease outbreaks	Five-point likert scale: 1. Not concerned … 5. Very concerned	0.933
	Beliefs about risks for human health *(B)*	6 items Large stables with many animals in particular are a health threat The emission of harmful substances is small in new, larger stables (reverse coded) The air around intensive livestock farms contains harmful substances The risk of infectious diseases passing from animals to humans is greater when animals are outside The closer you live to a livestock farm, the greater the risks for your health There is scientific evidence for the health risks of ILF	Five-point Likert scale: 1. Certainly not …. 5. Certainly	0.922
	Beliefs about ILF and odour/air quality *(B)*	3 items The air around ILF is highly polluted The smell of ILF is unhealthy The smell of ILF is a nuisance	Five-point Likert scale: 1. Certainly not …. 5. Certainly	0.916
	Beliefs about ILF and Q fever *(B)*	4 items Q fever is a problem in goat farms The Q fever bacterium can easily mutate The measures (milk control, compulsory vaccination of goats) are effective (reverse coded) There will be another Q fever outbreak within 10 years	Five-point Likert scale: 1. Certainly not …. 5. Certainly	0.784
	Beliefs about ILF and COVID-19 *(B)*	3 items People who live near ILF are more likely to get COVID-19 There is a relationship between air quality ILF and COVID-19 People who live near ILF get worse COVID-19 complaints	Five-point Likert scale: 1. Certainly not …. 5. Certainly	0.941
	Concerns about specific health hazards *(C)*	11 items To what extent are you concerned about: Antibiotic resistance Q fever African swine fever Air pollution (including particulate matter) Endotoxins COVID-19 Infectious diseases transmissible from animals to humans (including bird flu) Smell Sound Road safety Manure	Five-point likert scale: 1. Not concerned … 5. Very concerned	n.a.
Risks of ILF for animal welfare and nature	Beliefd that animals in ILF are well off in ILF *(B)*	4 items The animals in ILF are well cared for The health of the animals in ILF is good In the Netherlands, the animals in ILF are better off than in other countries The animals in ILF are now better off than before	Five-point Likert scale: 1. Certainly not …. 5. Certainly	0.915
	Beliefs about ILF harmfulness for nature *(B)*	2 items ILF damages nature ILF leads to less biodiversity	Five-point Likert scale: 1. Certainly not …. 5. Certainly	0.913
	Concerns about animals & nature *(C)*	6 items To what extent are you concerned about: Influence of manure on the environment Animal welfare Animal health Biodiversity Landscape Nitrogen emissions	Five-point likert scale: 1. Not concerned … 5. Very concerned	0.955
Risk mitigation and risk regulation	ILF is currently unsustainable *(O)*	9 items The Netherlands must stop the large-scale export of meat The livestock sector must switch to circular agriculture The livestock sector must switch to organic production The number of animals kept in a barn may increase further (reverse coding) ILF must be able to expand (reverse coding) ILF should be relocated to areas where there are no people living (i.e. industrial area) ILF is necessary to survive as a livestock farming business (reverse coding) Farmers with ILF will solve the biggest problems of ILF (reverse coding) The main problems of ILF can be solved with new technology (reverse coding)	Five-point Likert scale: 1. Disagree …. 5. Agree	0.908
	Local authorities favour farmers (*O)*	1 item My municipality gives too much weight to the interests of farmers with ILF	Five-point Likert scale: 1. Disagree …. 5. Agree 6. Don’t know^a^	n.a.
	Local authorities take concerns seriously *(O)*	3 items My municipality takes citizens’ concerns about ILF seriously My municipality takes complaints about ILF seriously My municipality communicates well about the expansion of livestock farms	Five-point Likert scale: 1. Disagree …. 5. Agree 6. Don’t know^a^	0.863
	Local authorities are trustworthy *(O)*	3 items My municipality’s rules and laws about ILF are sufficient My municipality is doing enough to enforce the rules for ILF My municipality adheres to agreements made about ILF	Five-point Likert scale: 1. Disagree …. 5. Agree 6. Don’t know^a^	0.878
	National authorities favour farmers *(O)*	1 item The national government gives too much weight to the interests of farmers with ILF	Five-point Likert scale: 1. Disagree …. 5. Agree	n.a.
			6. Don’t know^a^	
	National authorities take concerns seriously *(O)*	1 item The national government takes citizens’ concerns about ILF seriously	Five-point Likert scale: 1. Disagree …. 5. Agree	n.a.
			6. Don’t know^a^	
	National authorities are trustworthy *(O)*	3 items National government rules and laws on ILF are sufficient The national government is doing enough to enforce the rules for ILF The national government adheres to agreements made about ILF	Five-point Likert scale: 1. Disagree …. 5. Agree	0.741
			6. Don’t know^a^	

^a^Don’t know = analysed as missing.

The survey was pre-tested, whereby 13 respondents were asked to comment on its comprehensibility, difficulty and length. Based on the feedback, the surveys were further revised. The Medical Ethical Committee, VU University Medical Centre, reviewed the research protocol and determined that this research was not subject to Dutch law for medical research involving human subjects (WMO), and therefore concluded that it was exempt from seeking further approval from the Medical Ethical Committee.

### Data analyses

All data analysis were conducted with SPSS version 22.0. First, the two data sets were merged. After checking for irregularities, descriptives were generated. Principal component analyses (PCA) with Direct Oblimin rotation were then performed on conceptually interlinked items assessing the different themes that were identified in the previous qualitative study (Eijrond, Claassen and Timmermans 2022). Reliability analysis was conducted to ensure that the interlinked items could reliably be summed and averaged to form a internally consistent scale (and when deemed more consistent, items were reverse coded). Constructs were considered valid if the PCA indicated that the items within a constructs load on the same component, and when the internal consistency indicated by Cronbach’s alpha was 0.6 or higher (see Table 1). The differences in attitudes, beliefs, concerns and opinions between the general public in the research panel, farmers and other stakeholders in the stakeholder sample were explored by analysis of variances (One-way ANOVA). Differences were judged significant if *p* ≤ .05. An independent samples *t*-test was conducted in the research panel sample to determine whether people living in livestock dense municipalities hold different perspectives towards ILF compared to people living in other municipalities (see supplementary material).

## Results

### The study samples

In total 1412 people completed the survey; 808 were recruited through the research panel as a member of the general public; 274 were participants living in pre-selected livestock-dense municipalities in the South of the Netherlands. The remaining 534 respondents were sampled from other municipalities in the Netherlands. 604 respondents participated trough the external link of a stakeholder sample of famers and other stakeholders. As shown in Table 2, the combined samples included 238 participants that indicated that their main link to ILF was as a farmer (including one in the research panel), 285 participants that considered themselves neighbouring residents and 652 (concerned) citizens. The other 237 participants specified other links with ILF such as scientists/health professionals, policymakers and other. Due to the small sample size and heterogeneous groups, these groups will not be analysed separately. Completing the survey took approximately 25.9 min (research panel) and 29.6 min (stakeholder sample).

**Table 2.** Respondents’ characteristics (*n* = 1412).

	Research panel sample	Stakeholder sample		
	*n* = 808	*n* = 604		
	General public^a^	Farmers	Other stakeholders	Total
	*n* = 808 (%)	*n* = 237 (%)	*n* = 367 (%)	*n* = 1412 (%)
Stakeholders^b^				
Farmers	1	237	0	238
Neighbouring residents	105	0	180	285
(Concerned) citizens	565	0	87	652
Scientists & health professionals	17	0	22	39
Policymakers	1	0	12	13
Other^c^	119	0	66	185
Gender				
Male	430 (53.2)	157 (66.2)	267 (72.8)	854 (60.5)
Female	378 (46.8)	79 (33.3)	100 (27.2)	557 (39.5)
Other	0	1 (0.4)	0	1 (0.1)
Age				
18–34	159 (19.7)	49 (20.7)	67 (18.3)	275 (19.5)
35–44	108 (13.4)	44 (18.6)	84 (22.9)	236 (16.7)
45–54	123 (15.2)	91 (38.4)	126 (34.3)	340 (24.1)
55–64	165 (20.4)	44 (18.6)	72 (19.6)	281 (19.9)
65+	253 (31.4)	9 (3.8)	18 (4.9)	280 (19.8)
Education^d^				
Low	225 (27.9)	39 (16.5)	35 (9.5)	299 (21.2)
Middle	299 (37.0)	121 (51.1)	141 (38.4)	561 (39.7)
High	284 (35.1)	77 (32.5)	191 (52)	552 (39.1)
Health Status				
Excellent	108 (13.4)	154 (65.0)	173 (47.1)	435 (30.8)
Very good	192 (23.8)	56 (23.6)	104 (28.3)	352 (24.9)
Good	369 (45.7)	22 (9.3)	79 (21.5)	470 (33.3)
Satisfactory	111 (13.8)	3 (1.3)	9 (2.5)	123 (8.7)
Poor	26 (3.2)	1 (0.4)	1 (0.3)	28 (2)
(No comment)	2 (0.2)	1 (0.4)	1 (0.3)	4 (0.3)
Background^e^				
Friends, family/ acquaintances (have been) employed in the livestock sector	172 (21.3)	175 (73.8)	212 (57.8)	559 (39.6)
Frequently visits an area with livestock farms	117 (14.5)	160 (67.5)	180 (49.0)	457 (32.4)
Member of an animal/ environmental interest group	133 (16.5)	19 (8.0)	52 (14.2)	204 (14.4)
Raised in an area with livestock farms	241 (29.8)	190 (80.2)	260 (70.8)	691 (48.9)
Feels attached to the livestock sector	72 (9.0)	194 (81.9)	206 (56.1)	473 (33.5)
(Has been) employed in the livestock sector	24 (3.0)	186 (78.5)	128 (34.9)	338 (23.9)
Lives in an area with livestock farms	285 (35.3)	190 (80.2)	270 (73.6)	745 (52.8)
None of the above	280 (34.7)	0	8 (2.2)	288 (20.4)
Self–reported: living in a municipality with intensive livestock farms^f^				
Yes	259 (32.1)			
No	299 (37.0)			
Don’t know	250 (30.9)			

^a^Oversampling in research panel sample of 23 livestock-dense municipalities in the South of the Netherlands: Noord-Brabant: Someren, Alphen-Chaam, Sint Anthonis, Asten, Bernheze, Baarle-Nassau, Reusel de Mierden, Oirschot, Boekel, Boxmeer, Bergeijk, Eersel, Gemert Bakel, Deurne, Heeze-Leende, Zundert, Landerd, Mill en Sint Hubert. Limburg: Nederweert, Venray, Leudal, Peel en Maas, Weert, Horst aan de Maas (*n* = 274, 34% livestock-dense municipality and *n* = 534, 66% other municipalities)

^b^Self-reported, in response to the question: In what way are you most involved with intensive livestock farming in the Netherlands?

^c^Other include: member of an interest group, politician and family member, citizen, consumer, journalist, supplier, advisor or not applicable.

^d^Low: Primary education, prevocational secondary education (VMB), the first three years of senior general secondary education (HAVO) or pre-university secondary education (VWO) and the secondary vocational assistant’s training (MBO-1) Intermediate: Upper senior general or pre-university secondary education, basic vocational training (MBO-2), vocational training (MBO-3) and intermediate and specialist vocational training (MBO-4) High: University of applied sciences and research university education.

^e^More answers possible, so does not add to 100%.

^f^Self-reported, in response to the question: Do you live in a municipality with intensive livestock farming?

The characteristics of the respondents in the three samples, namely the general public (research panel sample), farmers and other stakeholders (stakeholder sample) are presented in Table 2. The majority of the respondents (in all three samples) was male, with a middle or high education level. Of the farmers in the stakeholder sample, the majority reported having pigs (38%) followed by cows (39%), poultry (23%) and goats (6%). Other animals included veal calves, sheep and horses. A majority of the farmers indicated having substantial (38%) or a lot (52%) of contact with citizens in their community.

#### Overall evaluation of ILF

Stakeholders’ evaluations towards ILF are depicted in Table 3. Participants in the research panel sample demonstrated relatively negative attitudes towards ILF and were concerned about ILF. In contrast, participating farmers had a positive attitude and were not concerned about ILF. Compared to farmers, the other stakeholders showed slightly less positive attitudes but did not differ significantly regarding concerns. Although all three groups had a positive opinion of farmers, participants in the research panel were the least positive. An independent-samples *t*-test showed that participants in the research panel living in livestock dense communities had a marginally significantly more positive attitude towards ILF (M: 3.30; SD: 1.03) compared to people living in other municipalities (M: 3.51, SD: 1.03), t(806) = 2.825, *p* = .005.

**Table 3.** Differences in attitudes, concerns and opinions towards ILF between stakeholders.

		Negative general attitude towards ILF (A)**	Concerned about ILF (C)***	Positive opinion of farmers (O)****
Sample	Stakeholders	Mean (SD)	Mean (SD)	Mean (SD)
Research panel sample	General public *N* = 808	3.44 (1.02)^b^^,c^	3.20 (1.54)^b,c^	3.11 (0.72)^b,c^
Stakeholder sample	Farmers *N* = 237	1.25 (0.41)^a^^,b^	1.36 (0.89)^a^	4.60 (0.42)^a^^,c^
	Other stakeholders *N* = 367	1.46 (0.85)^a^^,b^	1.51 (1.08)^a^	4.39 (0.72)^a^^,b^
ANOVA	*F*-value	F (2, 1409) = 917.559	F (2, 1409) = 293.660	F (2, 1409) = 706.975
	*p*-value*	*p* < .05	*p* < .05	*p* < .05

*p ≤ .05 significant difference.

**Scale: 5 items, e.g. positive – negative (higher scores indicate more negative attitudes).

***Scale: 1 (not concerned) to 5 (very concerned).

****Scale: 1 (disagree) to 5 (agree).

^a^Significant difference with research panel.

^b^Significant difference with farmers.

^c^Significant difference with other stakeholders.

#### Benefits and risks of ILF for society

As can be seen in Table 4, the participating members of the general public and farmers had significantly opposing opinions regarding the benefits and risks of ILF for society. Participants from the general public believed that production is predominantly for export purposes and only somewhat important for the Dutch economy. They also held strong beliefs that ILF is harmful for the living environment. In contrast, the belief that ILF is primarily associated with production for export was not supported by farmers. Also they considered ILF very important for the Dutch economy and strongly disagreed that ILF is harmful for the living environment. Although other stakeholders in the stakeholders sample did not differ significantly with farmers regarding the economic impacts, they were slightly more of the opinion that ILF is somewhat harmful for the living environment. *T*-tests showed no significant differences in mean scores between people living in livestock dense communities and other municipalities.

**Table 4.** Differences in beliefs and opinions of the benefits and risks of ILF for society.

		Production for export (B)***	Beneficial for the Dutch economy (O)**	Harmful for the living environment (O)**
Sample	Stakeholders	Mean (SD)	Mean (SD)	Mean (SD)
Research panel sample	General public *N* = 808	4.04 (0.80)^b,c^	3.01 (0.95)^b,c^	3.45 (0.98)^b,c^
Stakeholder sample	Farmers *N* = 237	2.43 (1.36)^a^	4.56 (0.56)^a^	1.43 (0.69)^a,c^
	Other stakeholders *N* = 367	2.49 (1.31)^a^	4.43 (0.82)^a^	1.76 (1.08)^a,b^
ANOVA	F-value	F (2, 1409) = 377.925	F (2, 1409) = 509.003	F (2, 1409) = 616.752
	*p*-value*	*p* < .05	*p* < .05	*p* < .05

*p ≤ .05 significant difference.

**Scale: 1 (disagree) to 5 (agree).

***Scale: 1 (certainly not) to 5 (certainly).

^a^Significant difference with research panel.

^b^Significant difference with farmers.

^c^Significant difference with other stakeholders.

#### Risks of ILF for human health & well-being

The general public in the research panel sample and farmers in the stakeholder sample demonstrated significantly different concerns and beliefs towards human health and ILF, as can be deduced from Table 5. On average, the general public sample was quite concerned about human health & wellbeing. Participants in this sample believed that ILF is a risk for human health. Odour/air quality and in particular Q fever and to a lesser extent COVID-19 are seen as health problems in relation to ILF. Their top three hazard concerns were antimicrobial resistant bacteria, followed by particulate matter and zoonoses. In contrast, participating farmers were not concerned about human health & wellbeing nor did they believe that ILF is a risk for human health. They also did not consider odour/air quality, Q fever and COVID-19 as problems relating to ILF. They were only slightly concerned about a specific health hazard i.e.swine flu. Other stakeholders in the stakeholder sample did not differ significantly with the farmers. In the *t*-test, significant differences were found in beliefs about odour/air quality and COVID-19. Participants living in livestock dense communities more strongly believed that odour/air quality was a health problem relating to ILF (M: 3.57, SD: 0.88) compared to people living in other municipalities (M: 3.39, SD: 0.79), t(806) = −2.939, *p* = .003. Also COVID-19 was characterised by participants living in livestock dense communities as more of an health issue related to ILF (M: 2.77, SD: 0.82) compared to people living in other municipalities (M: 2.61, SD: 0.89), t(806) = −2.546, *p* = .011.

**Table 5.** Differences in beliefs and concerns towards risks of ILF for human health & wellbeing between stakeholder groups.

		Concerns about human health & wellbeing (C)**	Risks for human health (B)***	Beliefs about ILF and Odour/air quality (B)***	Beliefs about ILF and Q-fever (B)***	Beliefs about ILF and COVID-19 (B)***	Top 3 concerns about human specific health hazards (C)**
Sample	Stakeholders	Mean (SD)	Mean (SD)	Mean (SD)	Mean (SD)	Mean (SD)	Mean (SD)
Research panel sample	General public *N* = 808	3.35 (0.99)^b,c^	3.42 (0.71)^b,c^	3.45 (0.82)^b,c^	3.54 (0.42)^b,c^	2.67 (0.87)^b,c^	1. Antimicrobial resistant bacteria: 3.63 (1.03) 2. Particulate Matter: 3.53 (1.07) 3. Zoonoses: 3.46 (0.99)
Stakeholder sample	Farmers *N* = 237	1.53 (0.52)^a^	1.49 (0.47)^a,c^	1.52 (0.61)^a,c^	2.88 (0.59)^a^	1.17 (0.38)^a,c^	1. Swine flu: 2.64 (1.26) 2. Q-fever: 1.77 (0.76) 3. Zoonoses: 1.76 (0.84)
	Other stakeholders *N* = 367	1.67 (0.90)^a^	1.71 (0.82)^a,b^	1.76 (0.96)^a,b^	2.87 (0.61)^a^	1.37 (0.74)^a,b^	1. Swine flu: 2.40 (1.23) 2. Q-fever: 1.82 (0.94) 3. Zoonoses: 1.77 (0.95)
ANOVA	F-value	F (2, 1409) = 638.072	F (2, 1409) = 1124.364	F (2, 1409) = 799.278	F (2, 1409) = 294.080	F (2, 1409) = 547.618	n.a.
	*p*-value*	*p* < .05	*p* < .05	*p* < .05	*p* < .05	*p* < .05	n.a.

*p ≤ .05 significant difference.

**Scale: 1 (not concerned) to 5 (very concerned).

***Scale: 1 (certainly not) to 5 (certainly).

^a^Significant difference with research panel.

^b^Significant difference with farmers.

^c^Significant difference with other stakeholders.

#### Risks of ILF for animal welfare and nature

As Table 6 illustrates, the participating members of the general public and farmers also had divergent beliefs about the risks for animals and nature. Although participants from the general public believed that animals are probably well off in ILF they also strongly believed that ILF is harmful for nature and indicated being very concerned about animals & nature. Farmers very strongly believed animals are well off in ILF and did not believe that ILF is harmful for nature and indicated being hardly concerned about animals & nature. Other participating stakeholders held similar views, yet compared to farmers they were somewhat more inclined to believe that ILF is harmful for nature and were slightly more concerned about animals and nature. People living in livestock dense communities, were significantly more positive regarding the treatment of animals in intensive livestock farms (M: 3.59, SD: 0.75) compared to people living in other communities (M: 3.29, SD: 0.82), t(806) = −5.118, *p* = .000.

**Table 6.** Differences in beliefs and concerns towards risks of ILF for animal welfare & nature and ILF between stakeholder groups.

		Animals are well of in ILF (B)**	Harmful for nature (B)**	Concerns about animals and nature (C)***
Sample	Stakeholders	Mean (SD)	Mean (SD)	Mean (SD)
Research panel sample	General public *N* = 808	3.39 (0.81)^b,c^	3.59 (0.91)^b.c^	3.41 (0.86)^b.c^
Stakeholder sample	Farmers *N* = 237	4.86 (0.27)^a^	1.57 (0.76)^a,c^	1.43 (0.48)^a,c^
	Other stakeholders *N* = 367	4.75 (0.54)^a^	1.86 (1.11)^a,b^	1.65 (0.87)^a,b^
ANOVA	F-value	F (2, 1409) = 734.831	F (2, 1409) = 664.222	F (2, 1409) = 891.079
	*p*-value*	*p* < .05	*p* < .05	*p* < .05

*p ≤ .05 significant difference.

**Scale: 1 (certainly not) to 5 (certainly).

***Scale: 1 (not concerned) to 5 (very concerned).

^a^Significant difference with research panel.

^b^Significant difference with farmers.

^c^Significant difference with other stakeholders.

#### Risk mitigation and risk regulation

The participating stakeholders’ opinions towards the future, local & national policy and ILF are presented in Table 7. The general public thought that ILF is currently unsustainable and agreed with solutions, such as switching to circular agriculture and/or to organic production. To some extent they also thought that the national authorities tend to favour farmers. There were no significant differences between people living in livestock dense communities and other citizens. The participants living in a municipality with intensive livestock farms believed that local authorities favour farmers but are somewhat positive towards the local authorities. In contrast, the participating farmers did not agree with the opinion that ILF is unsustainable nor did they think that national authorities place greater emphasis on the interest of farmers.

**Table 7.** Differences in opinions towards risk mitigation and risk regulation between stakeholder groups.

		Future	National policy			Local policy		
		ILF is currently unsustainable (O)**	NA favour farmers (O)***	NA take concerns seriously (O)***	NA are trustworthy (O)***	LA are favour farmers (O)***	LA take concerns seriously (O)***	LA trustworthy (O)***
Sample	Stakeholders	Mean (SD)	Mean (SD)	Mean (SD)	Mean (SD)	Mean (SD)	Mean (SD)	Mean (SD)
Research panel sample	General public *N* = 808/ local residents**** (*n* = 274)	3.40 (0.73)^b,c^	3.31 (1.08)^b,c^	3.09 (0.96)^b,c^	2.86 (0.82)^b,c^	3.32 (1.02)^b,c^	3.17 (0.85)^b,c^	3.13 (0.86)^b,c^
Stakeholder sample	Farmers *N* = 237	1.96 (0.50)^a^	1.96 (1.28)^a^	4.12 (0.90)^a^	3.96 (0.86)^a^	2.12 (1.12)^a^	4.18 (0.69)^a^	4.28 (0.72)^a^
	Other stakeholders *N* = 367	2.09 (0.72)^a^	1.90 (1.18)^a^	4.04 (1.01)^a^	3.91 (0.96)^a^	1.96 (1.02)^a^	4.12 (0.80)^a^	4.21 (0.82)^a^
ANOVA	F-value	F (2, 1409) = 668.018	F (2, 1332) = 242.227	F (2, 1300) = 166.320	F (2, 1319) = 250.401	F (2, 724) = 119.112	F (2, 745) = 124.201	F (2, 727) = 146.177
	*p*-value*	*p* < .05	*p* < .05	*p* < .05	*p* < .05	*p* < .05	*p* < .05	*p* < .05

*p ≤ .05 significant difference.

**Scale: 1 (disagree) to 5 (agree).

***Scale: 1 (disagree) to 5 (agree) and 6 (don’t know as missing).

****Only the respondents who identified themselves as living in a municipality with intensive livestock farms answered this question. As this is self-reported since people, it differs with the actual sample size of people living in one of the 23 preselected livestock dense communities.

^a^Significant difference with research panel.

^b^Significant difference with farmers.

^c^Significant difference with other stakeholders.

## Discussion

Our results show contrasting beliefs and concerns about the risks and benefits of ILF between the general public and farmers. The fairly negative attitude of the general public and the positive attitude among farmers are reflected in their views on the economic importance of ILF to society and their concerns in relation to human health, animals and nature. While the general public thinks ILF is unsustainable and changes are necessary, farmers do not share this opinion. People living in livestock dense communities held a more positive view towards ILF compared to people living in other communities, yet odour hinder and air quality was specifically perceived as a ILF related health problem. Our findings illustrate that differences in risk perceptions between farmers and the general public on a wide range of topics. To date, the differences in the multifaceted risk perceptions on ILF in the Netherlands has received scant attention in the research literature, as well as in policy making.

Previous studies about the public’s perceptions of the risks of ILF relate to specific topics such as zoonoses and antibacterial resistance (Stel, Eggers, and Nagelmann 2022; van Asselt et al. 2018; Verhue et al. 2011). However, public concerns remain even though antimicrobial usage has been reduced by more than 69% in livestock farming since 2009 in the Netherlands (Netherlands Veterinary Medicines Institute, 2021), and surveillance systems and vaccination programmes are used to detect and prevent potential threats of zoonotic livestock diseases. The public continues to be concerned, despite these top-down imposition of technical, or expertise-based solutions. It is likely that these concerns will not reduce due to recent zoonotic outbreaks such as the COVID-19 pandemic and worries regarding the spill over threat of highly pathogenic avian influenza. The prevalence of the large economic interests of the agricultural sector above the interests of public health e.g. during the Q fever outbreak (Haalboom 2017), affecting public trust in the government today may explain that despite the various measures against Q fever, it remains a point of concern among the general public.

The demonstrated divide between farmers and the general public regarding their attitude and concerns about ILF and its place in society further complements the findings from earlier more focused studies about animal welfare (Bokma-Bakker et al. 2011; Te Velde, Aarts, and Van Woerkum 2002; van Asselt 2019; Vanhonacker et al. 2008). These studies show that for the general public, animal welfare in livestock farms is a real concern (Clark et al. 2016; Verhue et al. 2011). In particular they seemed concerned for animals in very large (mega) stables as these are unnatural and poses a risk of infectious disease outbreaks and environmental waste (Bergstra, Gremmen, and Stassen 2015; Verhue et al. 2011). On the other hand, both previous as our own study also demonstrate that while farmers share the importance of the welfare of their animals they are not concerned about their health. One explanation is that they evaluate welfare differently. Farmers refer to aspects related to biological functioning emphasising freedom from disease and injury, food, water and shelter. Whereas citizens subscribe to the same values as farmers, they also emphasis the affective states referring to animals’ feelings and naturalness (Bergstra, Gremmen, and Stassen 2015; Cardoso, von Keyserlingk, and Hötzel 2019; Te Velde, Aarts, and Van Woerkum 2002; Vanhonacker et al. 2008).

Additional factors explaining why the general public and farmers differ in how they think about the risks and benefits of ILF may include knowledge, personal experiences, controllability of the perceived risk, confidence in risk reduction measures, interests, values and feelings. These are all factors shown to affect risk perceptions (Siegrist and Árvai 2020; Wachinger et al. 2013). For instance, farmers’ interests are predominantly economically driven, supplying quality products, having a satisfying job but also recapturing legitimacy in society (Te Velde, Aarts, and Van Woerkum 2002). Whereas the general publics’ interests may be easily available and affordable food from animals that were treated well (Te Velde, Aarts, and Van Woerkum 2002). Local residents may also have other interests such as living in a pleasant living environment. Farmers rely on their daily experience and practical experience (van Asselt et al. 2018; Vanhonacker et al. 2008), while citizens’ knowledge of the circumstances in which livestock is held is more limited (Vanhonacker et al. 2008). As a consequence, they assess risks and benefits of ILF differently and rely on indirect knowledge, e.g. media coverage. Media attention on zoonotic outbreaks such as Q fever and the COVID-19 pandemic as well as avian influenza, on barn fires and the nitrogen problem, may have amplified risk perceptions. The greater human health concerns of the general public as compared to farmers may also be related to having less personal control to mitigate the risks, which is also shown to be related to perceived risk (Slovic et al. 1982; van Asselt et al. 2018). Farmers also have a higher confidence in risk reduction measures (i.e. technology) as opposed to the public, hence have lower risk perceptions regarding human health of hazards such as zoonotic diseases and particulate matter (Eijrond, Claassen, and Timmermans 2022).

We found a few differences between the beliefs and concerns of the general public and nearby residents. Our earlier qualitative study showed that residents were not against livestock farming per se, but against the size, and preferred small-scale, family-owned, and biological farms. Residents are also more familiar with farming (Borlée et al. 2019). Nevertheless, our results also indicate that residents have concerns about certain aspects of livestock farming in their living area, in particular about odour and air quality. In contrast, farmers do not consider odour a big problem. Odour annoyance is a typical problem of livestock dense communities (Biesheuvel et al. 2019; Hooiveld et al. 2015; Post et al. 2020) and in general research has shown air quality is a health risk (Health Council of the Netherlands 2018a). Quite recently, large-scale epidemiological studies conducted in the Netherlands have found there is a higher risk of pneumonia for residents living in close proximity to goat farms, although there remain uncertainties about causal relationships (Hagenaars et al. 2017; Heederik et al. 2011; Ijzermans et al. 2018; Ijzermans et al. 2021; Maassen et al. 2016). Given the health risks associated with living in proximity to livestock farms and possible personal experiences with the Q fever epidemic, it would have been expected that individuals would be more concerned. Yet this is not the case. Proximity has been found earlier to be related to less concerns of nearby residents as compared to the general public (Bickerstaff and Simmons 2009; Sharp and Adua 2009; Sharp and Tucker 2005). There are a few possible explanations. Familiarity, social linkages, experience and economic motives are also associated with proximity and may reduce potential concerns. Alternatively, as residents chose to live near farms, they might have chosen to accept the risks due to the fact that the perceived benefits such as economic benefits, beauty of the landscape and calmness that outweigh the disadvantages of living in a rural area. Moreover, it is possible that they trust the authorities, experts as well as the farmers, by adopting risk mitigation measures such as vaccination of goats against Q fever (Wachinger et al. 2013).

Besides farmers and members of the general public, other stakeholder groups were also recruited in our study. This is a rather mixed group, i.e. newsletter members (consisting of interested researchers, health and environmental professionals, municipal officers, neighbouring residents and concerned citizens) of the consortium ‘Knowledge Platform Livestock and Human Health’. This consortium aims to make scientific information about the effects of livestock farming on human health available to professionals to help them in addressing questions and concerns of citizens and farmers (Knowledge Platform Livestock and Human Health, 2022). As the majority of this group indicated that their main link to ILF was as a resident or a concerned citizen, one would expect their beliefs and concerns to be more in agreement with the beliefs and concerns of the general public. Instead, we found that this stakeholder group perspectives were more in line with the positive view of the farmers. Such an alignment may not be that surprising as a majority in this group indicated to have strong positive connections in the livestock sector, e.g. being employed and/or having friends or family employed in the livestock sector.

In this paper, we addressed the perceptions of risk of many topics related to ILF across a diverse group of stakeholders. Yet, there are some limitations that we would like to highlight. The general public in the research panel sample was sampled to be representative of the larger population, but the participants may represent a group that may hold strong (negative or positive) views as participation was voluntary. The farmers were a convenience sample and therefore not sampled to be representative of all farmers. For instance, there may be differences between farmers with specific types of farms (i.e. conventional and organic). Although this study is limited to the Netherlands, we believe that some of these issues may also be relevant in areas with high animal and human population densities, such as in Belgium, Denmark, France and Germany (Robinson et al. 2011). However, these results should be extrapolated with caution, as there may be contextual differences, such as historical experiences, trust in the authorities and differences in livestock farming management.

Our study reflects more than just the perceived risks related to ILF, but also reveal the global and multidimensional legitimate concerns and views on what matter to different groups of people. There is a heterogeneity in perspectives on benefits and risks of ILF among the general public and citizens living in rural areas. These findings have multiple implications for risk communication and risk governance. First, health experts involved in science communication about the risks of ILF should be encouraged to pay attention to other (health) concerns that are relevant to the public. Experts involved are inclined to inform the public about their (health) risk assessment of intensive livestock farming often with the focus on one specific aspect that is considered a serious threat to health, e.g. the health risks of particulate matter. Information on issues experts believe are not a large problem (anymore) such as Q fever are rarely discussed (Eijrond et al. 2019). Moreover, odour may not be included in the communications as experts consider it only a minor health hazard compared to other hazards (Eijrond et al. 2019). In addition, by communicating only about, for instance, the impact on human health, citizens may feel they are not taken seriously as they often have multifaceted concerns. Second, the broader national debate about the future of farming should include discussion about the potential risks (e.g. zoonotic threats and environmental impact) of living in a livestock dense populated country as well as the benefits (e.g. the importance of the economy and affordable food).

It is perhaps not surprising that farmers and the general public have quite different perspectives about the sustainability of ILF. While the general public believes that ILF is no longer considered sustainable, farmers insist it is. In particular, the necessity of reduction of nitrogen emissions to stop the decline in biodiversity and comply with the European directive is strongly disputed by many farmers as they bear the brunt of the measures imposed to combat nitrogen emissions (Remkes et al. 2019). These differences therefore pose a genuine threat to the support for the necessary changes of ILF in the Netherlands. To come to terms with the differences in perspectives a more inclusive governance is inevitable. Scientific and technical expertise solving single problems alone cannot resolve the multifaceted problems, as these tend to overlook the complexity, the values, perceptions and lived experience of the stakeholders (Head 2008). It requires engaging all stakeholders in the risk evaluation and management process to come to a better mutual understanding and improve decision making (Renn 2021). As we found differences in perspectives between the general public and nearby residents in particular regarding odour, we argue that (some) local issues are different from national issues regarding ILF, and therefore need be approached differently by (local) policy makers. In conclusion, the contribution of this study is identifying and explaining the risk perceptions associated with ILF and the areas of conflict, which should aid policy makers with designing effective communication and integrating stakeholders’ perceptions in the design of morally acceptable risk management policies as well as determining the future direction of ILF in the Netherlands (Renn 2018).

## Supplementary Material

Supplemental Material

## Note

1 High human-livestock density combined with a low surface area (increasing the likelihood of local residents living close to livestock farms). Data on the number of inhabitants and number of livestock animals was gathered from Statistics Netherlands. Based on this data, the livestock density and human density was calculated in order to calculate the human-livestock density in every municipality.
