# Supplementary material for: Contrasting perspectives on the risks of intensive livestock farming in The Netherlands: a survey study
Source: J Risk Res. 2023 Jul 13;26(9):911–30. doi: 10.1080/13669877.2023.2231003 (PMC10561603; doi:10.1080/13669877.2023.2231003)
Supplement: Supplemental Material [file RJRR_A_2231003_SM3681.docx]

**Appendix**

Table 8. Independent samples t-test between results of residents of livestock dense municipalities and of other municipalities

|  | Livestock dense municipalities  N = 274 | | Other municipalities  N = 534 | | Independent-samples t-test | |
| --- | --- | --- | --- | --- | --- | --- |
|  | M | SD | M | SD | t-test | p-value* |
| General attitude towards ILF *(A)* | 3.30 | 1.03 | 3.51 | 1.03 | t (806) = 2.825 | **P = 0.005** |
| Concerned about ILF *(C)* | 3.09 | 1.37 | 3.26 | 1.62 | t (806) = 1.475 | P = 0.141 |
| Positive opinion of farmers *(O)* | 3.13 | 0.75 | 3.10 | 0.70 | t (806) =  -0.707 | P = 0.480 |
| Production for export *(B)* | 4.02 | 0.84 | 4.05 | 0.78 | t (806) = 0.478 | P = 0.633 |
| Important for the Dutch economy *(O)* | 3.06 | 0.96 | 2.98 | 0.94 | t (806) =  -1.181 | P = 0.238 |
| Harmful for the living environment (*O)* | 3.49 | 0.99 | 3.43 | 0.98 | t (806) =  -0.826 | P = 0.409 |
| Concerns about human health & wellbeing *(C)* | 3.38 | 1.01 | 3.33 | 0.98 | t (806) =  -0.730 | P =0.465 |
| Risks for human health *(B)* | 3.43 | 0.75 | 3.41 | 0.69 | t (806) =  -0.300 | P = 0.764 |
| Odour/air quality is a health issue *(B)* | 3.57 | 0.88 | 3.39 | 0.79 | t (806) =  -2.939 | **P = 0.03** |
| Q-fever is a health issue (*B)* | 3.56 | 0.44 | 3.52 | 0.41 | t (806) =  -1.178 | P = 0.239 |
| COVID-19 is a health issue *(B)* | 2.77 | 0.82 | 2.61 | 0.89 | t (806) =  -2.546 | **P = 0.011** |
| Animal wellbeing *(B)* | 4.56 | 0.57 | 4.66 | 0.49 | t (806) = 2.397 | **P = 0.017** |
| Animal health (*B)* | 4.57 | 0.52 | 4.63 | 0.47 | t (806) = 1.759 | P = 0.079 |
| Positive treatment of animals *(B)* | 3.59 | 0.75 | 3.29 | 0.82 | t (806) =  -5.118 | **P = 0.000** |
| Harmful for nature *(B)* | 3.59 | 0.88 | 3.59 | 0.92 | t (806) =  -0.035 | P = 0.972 |
| Concerns about animals and nature *(C)* | 3.36 | 0.88 | 3.44 | 0.86 | t (806) = 1.220 | P = 0.223 |
| Change is necessary *(O)* | 3.36 | 0.75 | 3.43 | 0.72 | t (806) = 1.289 | P = 0.198 |
| Communication needs to be improved *(O)**** | 3.96 | 0.71 | 3.90 | 0.69 | T(1409) =  -1.183 | P = 0.237 |
| NA favour farmers *(O)* | 3.34 | 1.07 | 3.29 | 1.08 | t (738) =  -0.687 | P = 0.493 |
| NA listen *(O)* | 3.03 | 0.94 | 3.12 | 0.97 | t (735) = 1.297 | P = 0.195 |
| NA are trustworthy (*O)* | 2.88 | 0.79 | 2.85 | 0.84 | t (723) =  -0.378 | P = 0.705 |

******* *p ≤ 0.05 significant difference*
